# Microbial cross contamination in household laundering and microbial ecology of household washing machines

**DOI:** 10.3389/fmicb.2025.1667606

**Published:** 2025-10-17

**Authors:** Kelly Whitehead, Jake Eppinger, Vanita Srinivasan, Juan A. Ugalde

**Affiliations:** ^1^Global Research and Development for Lysol and Dettol, R&D Microbiology and Virology Department, Reckitt Benckiser LLC, Montvale, NJ, United States; ^2^Center for Bioinformatics and Integrative Biology, Facultad de Ciencias de la Vida, Universidad Andres Bello, Santiago, Chile

**Keywords:** laundry, washing machines, microbiome, cross-contamination, biofilm, opportunistic pathogens, household hygiene

## Abstract

Household washing machines host diverse microbial communities that may include opportunistic pathogens, potentially impacting laundry hygiene and human health. However, our understanding of these communities and their transfer abilities remains limited. We examined microbial communities from 10 household washing machines (five front-load and five top-load) using surface swabs from specific hotspots and sterile sentinel washcloths. Samples were analyzed using culture-based methods and 16S rRNA/ITS metabarcoding. We tested microbial transfer during washing cycles with and without clothing and evaluated the effects of machine drying on this transfer. Front-load machines had significantly higher microbial loads than top-load machines (average bacterial counts: 6.50 ± 2.46 Log10/swab vs. 3.79 ± 1.73 Log10/swab). The microbial community composition was mainly shaped by the machine user rather than the machine type or sampling location. The dominant bacterial genera included *Pseudomonas*, *Micrococcus*, and *Sphingomonas*, while *Aspergillus*, *Cladosporium*, and *Penicillium* dominated the fungal communities. Opportunistic microorganisms were identified, but no highly pathogenic species (pathogenicity score 3) were found. Machine drying did not significantly decrease microbial loads, whereas the presence of soiled clothing impacted community composition. Household washing machines host user-specific microbial communities, including potential opportunistic pathogens. Current laundry practices may be inadequate for the complete elimination of pathogens, especially in immunocompromised individuals. These results support the need for additional household laundry sanitization strategies.

## 1 Introduction

Household washing machines, present in over 80% of homes in developed countries, play a crucial role in maintaining hygiene and preventing disease transmission. However, these appliances can paradoxically serve as reservoirs for diverse microbial communities, including potential pathogens, with significant public health implications (Satari et al., 2023). Washing machines are among the most prevalent household appliances worldwide. Washing machine ownership exceeds 80% in most developed countries, making these appliances ubiquitous in modern households and essential for daily hygiene practices (Washing Machines - Statistics and Facts, n.d.). Household laundry has become an integral aspect of modern life and is essential for maintaining personal hygiene and cleanliness standards. In contrast to the vital role that washing machines play in human hygiene practices, they provide an ideal environment for the growth of microorganisms, such as bacteria and fungi, across a range of temperatures, moist environments, and pH values. This persistence is partly attributable to the presence of nutrients derived from the chemical compounds used for washing and the introduction of microorganisms from the clothing (Abney et al., 2021).

Recent studies have shown that washing machines can contain various microorganisms (Callewaert et al., 2015; Nix et al., 2015; Jacksch et al., 2019, 2021; Whitehead et al., 2022; Cao et al., 2024; Chen et al., 2024). The structure of these microbial communities can be influenced by multiple factors, including the type of machine, frequency of use, usage (commercial versus domestic), water source, and user habits (Jacksch et al., 2019). For instance, front-load, agitator, and impeller top-load washing machines have distinct configurations that create unique areas where moisture and debris can accumulate, providing an ideal environment for microbial growth. Moreover, user habits, such as the type of detergent used or the frequency of machine cleaning, can significantly impact the types and abundance of microorganisms found in these appliances, even within the same geographical location (Abney et al., 2021). Clothing can serve as a transmission vehicle for these microorganisms, allowing them to move from human skin and exposed environments into washing machines and be transferred between washing cycles.

Some of the most prevalent microorganisms in washing machines belong to the genera *Pseudomonas, Acinetobacter*, and *Enhydrobacte*r (Jacksch et al., 2019; Satari et al., 2023). These organisms can originate from both human sources and the environment, with primary sources including air, soil, and tap water (Jacksch et al., 2019; Schmithausen et al., 2019). The presence of opportunistic microorganisms in various areas of washing machines is a major concern. Recent studies have identified specific bacterial genera, such as *Pseudomonas, Escherichia*, and *Acinetobacter*, and fungal genera, such as *Candida, Cryptococcus*, and *Rhodotorula*, as potential opportunistic microorganisms found in washing machines (Bockmühl et al., 2019; Chen et al., 2024). These microorganisms can potentially transfer and contaminate the laundry, increasing the risk of infection spread within residential and healthcare settings (Michael et al., 2017; Reynolds et al., 2022; Jung et al., 2023).

Unlike washing machines, research on microorganisms present on the surface and interior of dryers is limited to a few studies. Studies have examined the effects of air drying and temperature on the survival of fungal and bacterial pathogens, as well as enteric viruses (Walter and Schillinger, 1975; Gerba and Kennedy, 2007), showing how various temperatures affect the survival of different pathogenic bacteria (Shin et al., 2020). However, to the best of our knowledge, no study has explored the impact of drying on the microbial composition of laundered clothing.

Despite increasing research, significant gaps remain in our understanding of the microbiome of washing machines in household environments. The complexity of microbial communities, including the presence of opportunistic microorganisms influenced by various environmental factors and human activity, presents a challenge in defining a healthy or typical microbial community in household appliances. Addressing these challenges and increasing our understanding of the microbial communities within washing machines and dryers, as well as the potential exposure to pathogens during laundering, can provide valuable insights into the role of microorganisms in our laundry hygiene practices.

Despite growing research interest, key gaps remain, such as (1) detailed profiling of washing machine microbiomes across different machine types and user behaviors, (2) the potential for microbial transfer between machine surfaces and laundry items, and (3) the effectiveness of standard washing methods in removing possible pathogens. Understanding these aspects is vital for developing evidence-based household laundry hygiene guidelines, particularly for vulnerable populations. In this study, we examined the microbiome composition of washing machines using 16S rRNA and ITS metabarcoding with two sampling methods. The first method targeted microbial hotspots in both top-loading and front-loading machines in homes, providing a comprehensive view of microbial diversity, bioburden levels, and potential pathogens. We also investigated how machine design and usage influenced the microbiome profiles. The second method evaluated the impact of washing cycles by analyzing microbial transfer: (1) between machine surfaces and sterile items, and (2) among surfaces, sterile items, and typical soiled laundry during washing and drying. We hypothesized that household washing machines contain diverse, user-specific microbial communities influenced by machine design and usage patterns, and that these communities can be transferred between machine surfaces and laundry items during typical washing and drying processes.

## 2 Materials and methods

### 2.1 Sample collection, viable plate counts, and processing for DNA extraction

#### 2.1.1 Surface swab

Ten household washing machines (five front-load and five top-load) were recruited from volunteers in New Jersey and New York through convenience sampling. Surface swab sampling was conducted from predetermined hotspots within each washing machine, selected based on their proximity to the wash chamber, contact with detergents/softeners or influent water, and their propensity to retain moisture. Swabbing locations differed between top- and front-load machines, with five swab points for top-load models and six swab points for front-load models. All households used standard commercial detergent. While detergents can reduce microbial loads, they are not sterilizing agents, and microorganisms can persist through laundering, particularly in biofilms on machine surfaces or in niches where detergent penetration is limited.

The locations for swabbing within a front-loading washer were as follows: (1) internal drum wall, (2) rubber door seal, (3) water spray ports, (4) drum lower door edge (contact point with the lower gasket), (5) detergent drawer area, and (6) detergent drawer water spray port. The locations for swabbing within a top-loading washer were (1) inside the fabric softener reservoir area, (2) internal drum wall (near the top edge), (3) gasket/area around the (top) rim of the drum, (4) bottom drum area (with a focus on the corners), and (5) mixer/agitator spindle. Each location was swabbed using a sterile swab wetted with 1x phosphate-buffered saline (PBS) (Becton Dickinson GmbH, Heidelberg, Germany). A 2″ × 2″ sampling template/guide and a multi-directional zig-zag pattern were utilized for the internal drum (top and front loading), drawer area (front loading only), and bottom area (top-loading only). The rubber door seal and bottom door edge of the front-loading machines, along with the gasket/area around the rim of the top-loading machines, were sampled along a “half-moon” pattern. After sampling, swabs were placed in 2.0 mL of PBS with three sterile glass beads and processed for Total Viable Counts (TVC) using standard microbiological techniques (and described incubation conditions) as well as processed for molecular analysis (as described).

#### 2.1.2 Washcloth sampling

Identical cotton washcloths measuring 12 inches × 12 inches (Room Essentials, Target) were used in this assay. First, the washcloths were washed/dried (using standard washing machine cold and drying cycles). They were then autoclaved (Steris Amsco Lab Autoclave 250, San Diego, CA, United States) using a standard dry/gravity sterilization autoclave cycle in sealed autoclave bags [Medline Sterilization Pouches (12″ × 15″)]. Sterilized sentinel washcloths were subjected to two experimental conditions: (1) Run 1 - washcloths washed alone with standard liquid detergent (no bleach or sanitizer), and (2) Run 2 - washcloths washed together with typical soiled household laundry (“whites”). Two washcloths were used per load in each condition across five front-load and five top-load machines (each of the washing machines utilized the same type/lot of liquid detergent). The selection of machines is expected to reflect the consumer machines commonly found in households, considering the users’ regular cleaning habits. No specific selection of machines was conducted; the owners were asked not to clean their machines before the study period. The age of the machines utilized in this study was approximately 3–20 years (washing machines) and 8–20 years (dryers). A questionnaire covering the make, model, age, and usage habits (including how often units are in use, unit cleaning habits, wash cycle utilization, and routinely used products) was provided to each household.

For each run, a short, cold-water wash cycle (run 1) or an appropriate setting for the load size (run 2) was used with either a small load consumer detergent sample volume (run 1) or a medium load consumer detergent sample volume (run 2, 2–4 indentation level on the detergent cap, approximately 50–80 mL). The average water temperature across all washes was 13.98 °C. The documented wash time was determined to range from 9 min, 40 s to 33 min, 10 s (run 1) and 18 min, 10 s to 40 min, 16 s (run 2). For each load from each household washing machine, one of the sentinel washcloths was retrieved wet after washing using aseptic sampling measures, and the other washcloth was placed (alone) in the dryer for 30 min at a high heat setting (the average dryer temperature was determined to be 56.53 °C). The samples were returned to the laboratory while still moist or after drying for processing. No more than 24 h elapsed between sampling and processing. The sentinel washcloths were stored overnight at 2–8 °C when not processed immediately.

### 2.2 Recovery of microbial bioburden load and total viable counting procedures

Sentinel (dry and wet) washcloths were individually placed in sterile Whirl-Pak Stomacher bags containing 200 mL of PBS and processed in a Seward Stomacher at 180–260 rpm for 10 min. Expressed liquid samples (one per sentinel washcloth, whether washed with or without clothing or dried or not dried) were plated in duplicate for total viable bacteria on Tryptic Soy Agar and incubated at 35–37 °C for at least 48 h. These media were selected as general-purpose broad-spectrum media commonly used to recover a wide range of environmental bacteria and fungi. To quantify the total viable fungi in the samples, Hardy Malt Extract Agar (with chloramphenicol) was used, and the inoculated plates were incubated at 29–31 °C for at least 10 days. The use of different incubation temperatures for bacteria and fungi was based on the preferences of the two microorganism types at higher or lower temperatures, respectively. Our goal was to isolate viable microorganisms and not duplicate the conditions in which washing machine microbiomes might exist. Statistical analyses were performed using R (version 4.0.0). The normality of the data was assessed using the Shapiro–Wilk test. For normally distributed data, two-sample t-tests were used, and for non-normally distributed data, Wilcoxon rank-sum tests were applied. Statistical significance was set at α = 0.05. These procedures allowed the recovery of viable organisms even after drying, although isolates were not further identified using other methods, only used for the TVC.

### 2.3 Sample processing for DNA extraction

After plating, the liquid samples (one per sentinel washcloth or one for each swab) were centrifuged at 10,000 × g for 15 min in a Labnet Prism microcentrifuge. Multiple microcentrifuge runs were performed for each washcloth liquid sample, with subsequent pooling of the resulting pellets to compensate for significant sample volumes and optimize the recovery of genetic material for identification. The final pooled pellets were resuspended in approximately 1 mL DNA/RNA Shield solution (Zymo Research, Irvine, CA, United States) and sent to Zymo for metabarcoding analyses.

### 2.4 Sample metabarcoding

The samples were submitted to Zymo Research for metabarcoding using the ZymoBIOMICS Targeted Sequencing Service (Zymo Research, Irving, CA, United States). For most samples, the ZymoBIOMICS DNA Miniprep Kit (Zymo Research, Irvine, CA, United States) was used, whereas for low-biomass samples, the ZymoBIOMICS DNA Microprep Kit (Zymo Research, Irvine, CA, United States) was used.

Metabarcoding of the 16S rRNA gene was performed using the Quick-16S NGS Library Prep Kit (Zymo Research, Irvine, CA, United States), targeting the V3-V4 region of the gene. Fungal ITS was sequenced using the Quick-16S NGS library Prep Kit with primers targeting the ITS2 region. A negative control (blank extraction control) was included to account for possible contamination from the laboratory sampling process. Sequencing was performed on an Illumina MiSeq (Illumina, San Diego, CA, United States) using the V3 reagent kit (600 cycles, 2 × 301 paired-end reads), with the addition of >10% PhiX spike-in to generate library diversity.

### 2.5 Bioinformatic and statistical analysis

Zymo Research performed the initial bioinformatic analysis. Sequences were filtered using the DADA2 workflow (Callahan et al., 2016) to generate amplicon sequencing variants (ASVs), including the removal of sequencing errors. Chimeric sequences were removed using DADA2. Taxonomic assignments were performed using Uclust in QIIME v.1.9.1 (Caporaso et al., 2010) against the Zymo Research Database, a curated reference database developed by Zymo Research.

Further analysis included the removal of negative controls from the ASV tables. Samples with fewer than 1,000 reads were also removed from the study, as were ASVs with no taxonomic assignments at either the Kingdom or Phylum level and ASVs assigned to organelles. The removal of ASVs from the negative controls allowed us to account for background contamination.

The relative abundance composition was visualized using *phyloseq* (McMurdie and Holmes, 2013) and *microViz* (Barnett et al., 2021). For alpha diversity analysis, samples were rarefied to the minimum number of read counts for each dataset (surface swabs: 7,646 reads for 16S and 2,234 for ITS; washcloths: 7,814 reads for 16S and 3,619 reads for washcloths) using *phyloseq*, and visualized using *ggstatsplot* (Patil, 2021), where statistical differences were evaluated using the Mann–Whitney test as implemented in *ggstatsplot*. Beta diversity was performed on the log-transformed read counts using the Bray-Curtis distance and visualized on a PCoA plot using *phyloseq* (McMurdie and Holmes, 2013). We performed a PERMANOVA analysis using the *adonis* function implemented in the *vegan* package (Dixon, 2003) to evaluate the differences between groups.

We aimed to examine the specific types and categories of microorganisms present in the washing machine microbiome in greater detail, focusing on opportunistic pathogens. To accomplish this, we selected ASVs that were unambiguously assigned to particular bacterial or fungal species. This classification was then compared to a database that categorizes microorganisms based on their pathogenic potential (Sierra et al., 2019) and assigns a score associated with the likelihood of the microorganism being a pathogen. A score of 1 indicates that it is very unlikely to be a pathogen, 2 potential pathogens, and 3 very likely to be pathogens. Upon examination of the surface swab results, no bacterial or fungal taxa with a pathogenic score of 3 were detected, suggesting that, based on these results, no pathogenic taxa were sampled in the surface swabs.

## 3 Results

### 3.1 Total viable counts

Surface swabs from microbial hotspots revealed significant differences in microbial loads between the machine types (Tables 1, 2). In top-load machines, the highest bacterial recovery was observed at the drum gasket (3.79 ± 1.73 log₁₀ CFU/swab), which also showed the highest fungal counts (2.27 ± 0.87 log₁₀ CFU/swab). Front-load machines exhibited higher overall microbial loads, with door seals and detergent drawers showing bacterial counts of up to 6.50 ± 2.46 log₁₀ CFU/swab. Overall, front-load machines showed a higher microbial load than top-load ones.

**Table 1:** Bacterial and fungal viable counts from surface swabs of top-load washing machines (n = 5).

Swab location	Mean bacterial count	Mean fungal count
Top-load washing machine swab recoveries (log₁₀ CFU/swab)		
Fabric softener reservoir	2.46 ± 1.49	1.30 ± 0
Drum wall	3.71 ± 1.02	1.53 ± 0.34
Drum gasket	3.79 ± 1.73	2.27 ± 0.87
Drum lower edge	2.05 ± 0.9	1.27 ± 0.07
Agitator spindle	2.68 ± 0.36	1.1 ± 0.5

**Table 2:** Bacterial and fungal viable counts from surface swabs of front-load washing machines (*n* = 5).

Swab location	Mean bacterial count	Mean fungal count
Front-load washing machine swab recoveries (log₁₀ CFU/swab)		
Drum wall	3.32 ± 1.44	1.68 ± 0.53
Door seal	6.50 ± 2.46	3.25 ± 1.4
Water spray port	4.90 ± 2.94	2.48 ± 1.64
Drum lower edge	5.02 ± 2.67	2.39 ± 1.11
Detergent drawer	4.71 + 2.80	2.87 ± 1.63
Detergent drawer water port	6.28 ± 2.54	3.63 ± 1.35

Sentinel washcloths were used to evaluate the microbial bioburden in each of the sampled washing machines, comparing the outcome of washing with or without clothes and the effect of the drying cycle on the microbial count. The results (Tables 3, 4) showed that drying did not significantly reduce bacterial or fungal recovery, and no significant changes were observed upon the introduction of soiled clothing items.

**Table 3:** Bacterial and fungal plate counts in washcloth samples.

Sample	Mean bacterial count	Mean fungal count
Washcloth wet vs dry recoveries (log₁₀ CFU/washcloth)		
Run 1 (no clothing) wet sample	6.59 ± 1.08	3.36 ± 0.94
Run 1 (no clothing) dry sample	5.95 ± 1.23	3.47 ± 0.84
Run 2 wet sample (with consumer clothing)	6.42 ± 1.28	3.03 ± 1.07
Run 2 dry sample (with consumer clothing)	6.10 ± 1.36	3.13 ± 0.99

**Table 4:** Statistical evaluation of bacterial and fungal counts in dry versus wet washcloths.

Comparison	Test	*p*-value
Bacterial counts, dry vs. wet	Wilcoxon	0.143
Fungal counts, dry vs. wet	Two-sample *t*-test	0.789
Bacterial counts, dry vs. wet (with consumer clothing)	Two-sample *t*-test	0.595
Bacterial counts, dry vs. wet (with consumer clothing)	Two-sample *t*-test	0.827

### 3.2 Microbial community composition

#### 3.2.1 Surface swabs

**Figure 1:**
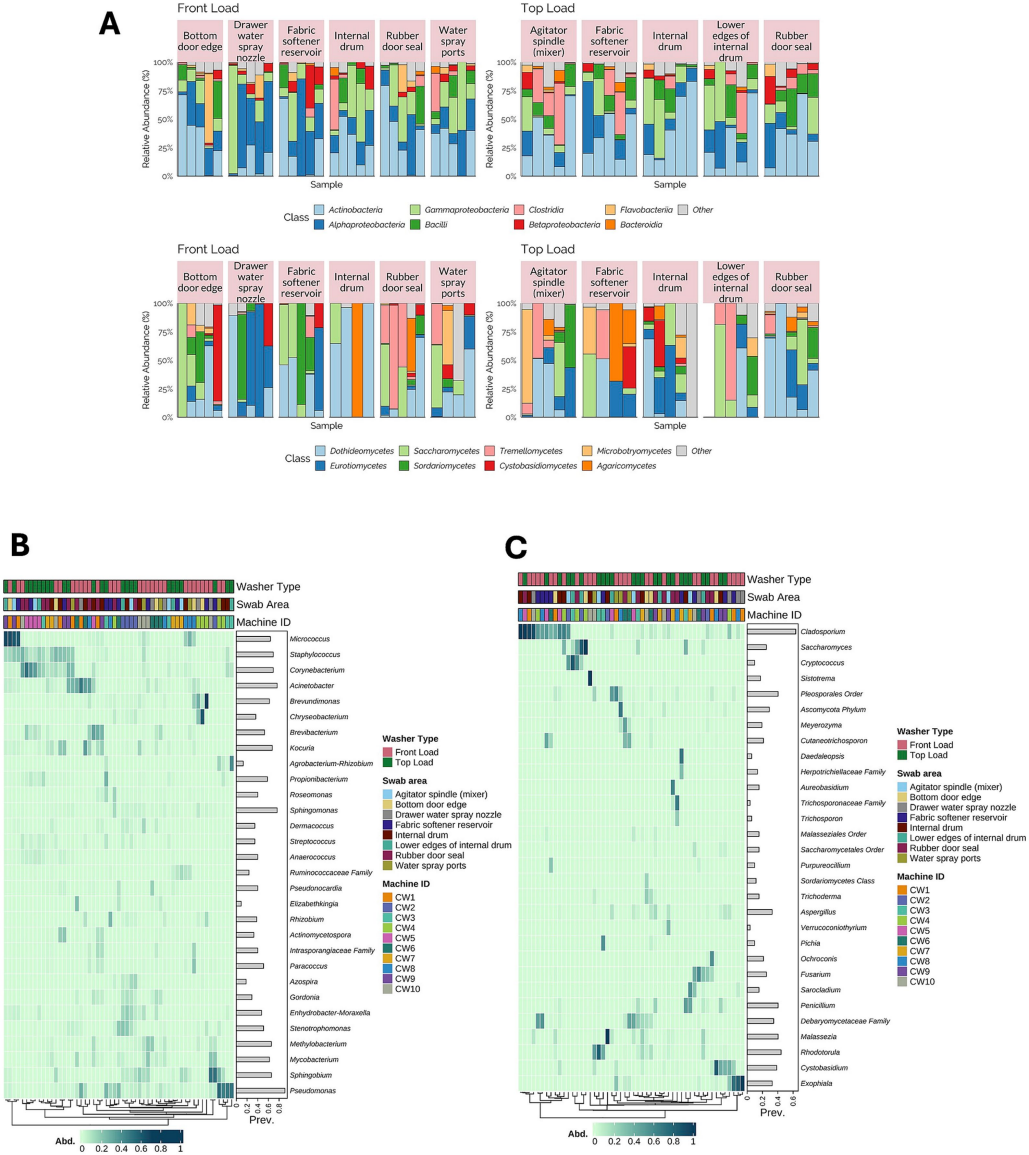
Microbial community composition in washing machine surface swabs. **(A)** Relative abundance of bacterial and fungal taxa at the class level across front-load (*n* = 30) and top-load (*n* = 25) machines and sampling locations, showing that the microbial profiles vary by washer type and site. **(B)** Hierarchical clustering heatmap of bacterial genera in surface swabs (log-transformed relative abundances), illustrating the distinct bacterial communities associated with different machine types and areas. **(C)** Hierarchical clustering heatmap of fungal genera in surface swabs, revealing the variable colonization of surfaces by opportunistic fungal taxa.

After processing the surface swab dataset, 3,683 ASVs and 55 samples were used for bacterial analysis, and 1,030 ASVs and 52 samples were used for fungal analysis. Initially, we assessed the microbial community composition at the class level for both bacterial and fungal groups across different machine types and sampling locations (Figure 1A). We observed considerable variation in bacterial composition among individual swab samples and swabbed areas, with no apparent dissimilarities between front- and top-load washing machines. The detected microbial communities were composed of microorganisms found in environments with high human activity and intervention, such as members of Gammaproteobacteria and Alphaproteobacteria, and environmental microorganisms such as Actinobacteria. For fungal communities, we observed considerable variation among samples and surfaces, with diverse taxa in all samples.

Further examination at the genus level revealed variations among taxonomic groups within the samples. Bacterial communities exhibited a high prevalence of taxa, including *Pseudomonas, Micrococcus, Sphingomonas,* and *Staphylococcus* (Figure 1B). These organisms are typically associated with human activities and are commonly found in the skin microbiome (Byrd et al., 2018). Several of these taxa were present in a significant fraction of the sampled machines but were dominant in only a few. However, the samples were clustered based on the owners of the sampled washing machines. For instance, *Pseudomonas* was prevalent in all samples, with a higher abundance in CW3 (front load) and CW4 (top load) than in other machines.

We observed a high prevalence of mold-related taxa, including *Aspergillus*, *Cladosporium*, *Penicillium*, and *Trichoderma* in the evaluated fungal communities (Figure 1C). These taxa were present in low abundance in most samples, but their abundance was higher in specific areas, such as the internal drum, rubber door seal, and fabric softener swabs from front-load washing machines.

#### 3.2.2 Washcloths

**Figure 2:**
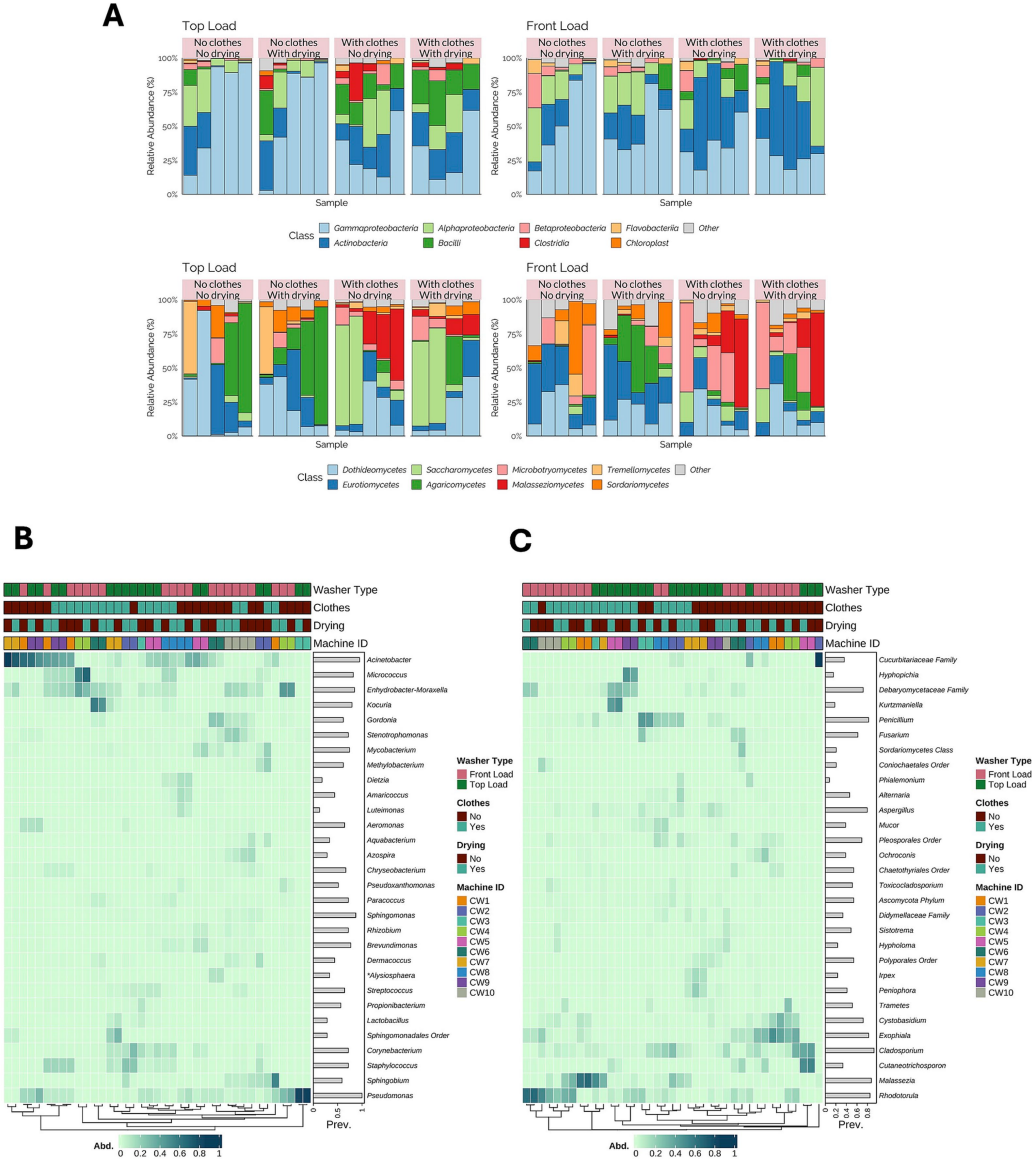
Microbial community composition of washcloth samples. **(A)** Relative abundance of bacterial and fungal taxa at the class level in washcloths, stratified by washer type, presence of clothes, and drying conditions, revealed that these factors significantly influenced microbial load and composition. **(B)** Hierarchical clustering heatmap of bacterial genera in washcloths, showing clustering patterns influenced by the washer type, presence of clothes, and drying. **(C)** Hierarchical clustering heatmap of fungal genera in washcloths, highlighting the differences in fungal colonization across machine conditions.

For the washcloth dataset, 2,315 ASVs and 39 samples were included in the bacterial analysis, and 2,165 ASVs and 39 samples were included in the fungal analysis. The bacterial community composition showed variations in the relative abundance of different bacterial classes, which were influenced by the presence of soiled laundry during the washing cycle (Figure 2A). For example, in the samples where clothes were present in the washing cycle, *Clostridia* was more abundant in some of the samples than when no clothes were present. Gammaproteobacteria, Actinobacteria, and Alphaproteobacteria were the dominant groups in the washcloth samples. Furthermore, we observed that including clothes during the washing cycle influenced the composition of the bacterial communities compared to the distribution observed in the samples where a drying cycle was used. The same effect was observed in fungal communities (Figure 2A), where the inclusion of clothes increased the relative abundance of the Class Malasseziomycetes.

When comparing the relative abundance of bacterial genera, *Pseudomonas*, *Sphingomonas*, and *Acinetobacter* were the most prevalent taxa in the washcloth samples (Figure 2B). However, we observed some clustering of the samples based on the machine used (Machine ID) and whether the washcloth was washed in the presence or absence of clothes, whereas drying did not appear to influence the bacterial community composition.

In the case of fungal communities, we observed clustering of the samples based on the origin of the sample, either front- or top-load washing machine (Figure 2C). The genera *Rhodotorula*, *Malassezia*, and *Penicillium* dominated fungal communities. Similar to what was observed with the bacterial communities, the inclusion of clothes during the laundering process appeared to influence the composition of the fungal communities compared to the inclusion of a drying cycle.

### 3.3 Microbial diversity

#### 3.3.1 Alpha diversity

**Figure 3:**
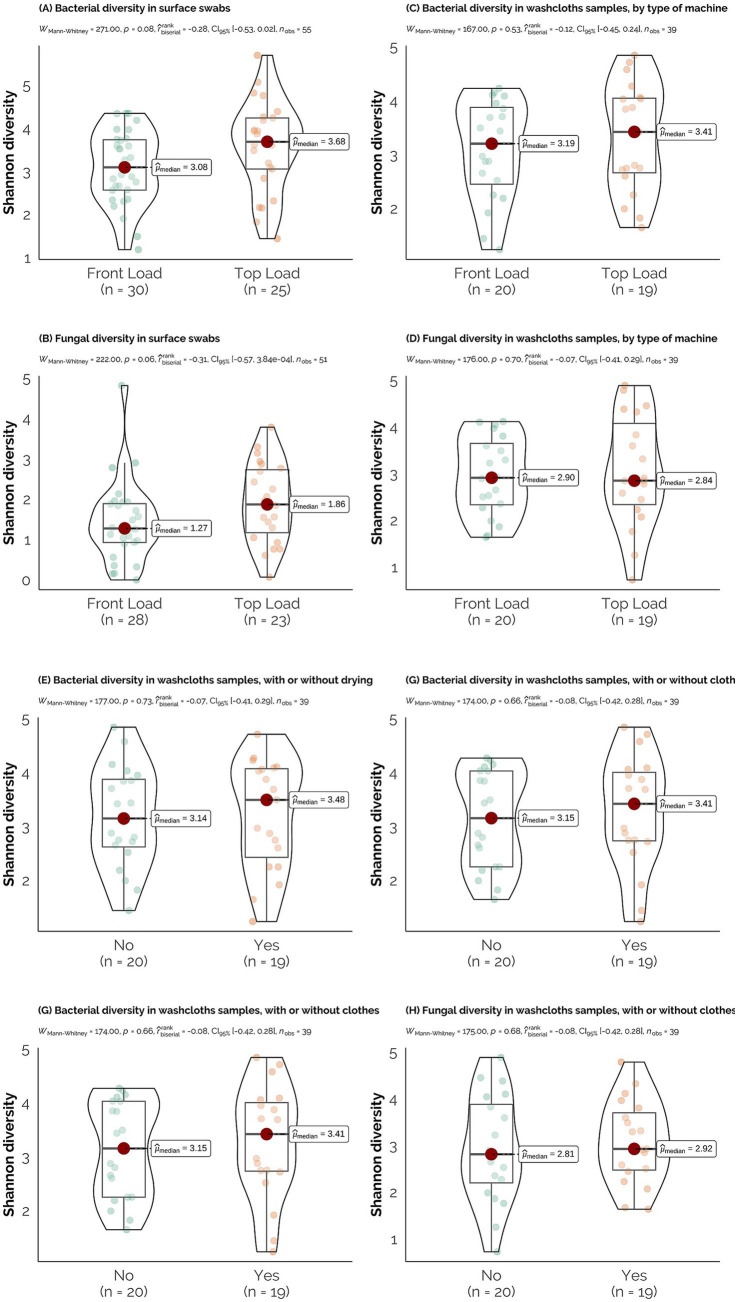
Alpha diversity analysis of the microbial communities. **(A,B)** Shannon diversity index for bacterial and fungal communities in surface swabs, indicating differences in diversity between front-load and top-load washing machines. **(C–H)** Shannon diversity of bacterial and fungal communities in washcloths, showing how washer type, drying, and presence of clothes influence community diversity.

We evaluated whether the type of machine (top-versus front-load) had an impact on the microbial diversity (bacterial and fungal) of the sampled areas, using the Shannon diversity index (Figures 3A,B). The results revealed no significant differences in the Shannon index across machine types. Furthermore, no significant differences were observed when comparing the swabbed areas (Supplementary Figure S1).

Additionally, we examined whether the type of machine influenced the microbial diversity of the washcloth samples (Figures 3C,D). Similar to the surface swabs, our analysis revealed no significant difference based on the type of machine used. As the washcloth experiment also included comparisons with the addition of clothes to the washing cycle and a drying step, we performed different comparisons to determine whether drying (Figures 3E,F) or the incorporation of clothes (Figures 3G,H) impacted microbial diversity. Consistent with previous observations, no disparities in alpha diversity were found when all treatments were examined.

### 3.4 Beta diversity

**Figure 4:**
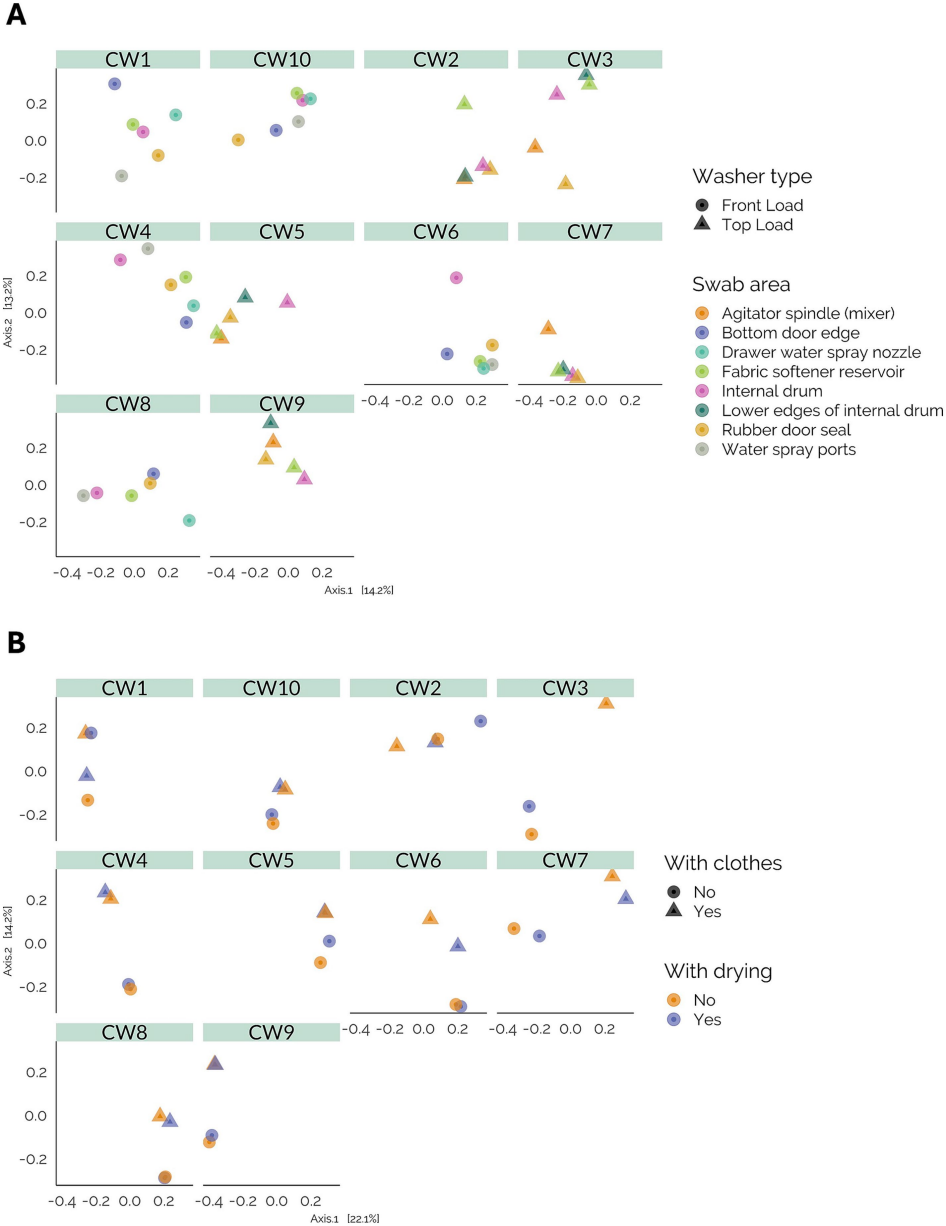
Beta diversity analysis. **(A)** Principal coordinates analysis (PCoA) of bacterial communities in surface swabs based on Bray–Curtis dissimilarity, demonstrating clustering of samples by machine and surface type. **(B)** Principal coordinates analysis (PCoA) of bacterial communities in washcloths, highlighting the effects of drying and clothes on community composition.

To assess the similarity of the microbial communities across the sampled areas with the surface swabs, we performed a beta diversity analysis comparing swabbed areas, machine type, and sample origin (Figure 4A). No evident clustering of samples was observed based on the swabbed location within each machine. However, a statistically significant separation between machine types (front-load versus top-load) was observed (*p* = 0.001). In addition, when we compared the machine ID, we found statistically significant differences (*p* = 0.003), suggesting that the differences in microbial composition were related to the user of the machine.

For the washcloth samples, the analysis revealed differences in the microbial composition based on the type of machine (*p* = 0.001) and the machine ID (*p* = 0.001) (Figure 4B). When comparing the addition of a drying cycle to the process, we did not observe a significant alteration in the microbial community composition (*p* = 1). However, the addition of clothes changed the microbial composition, with differences between the two groups (*p* = 0.003).

### 3.5 Presence of potentially pathogenic microorganisms

**Figure 5:**
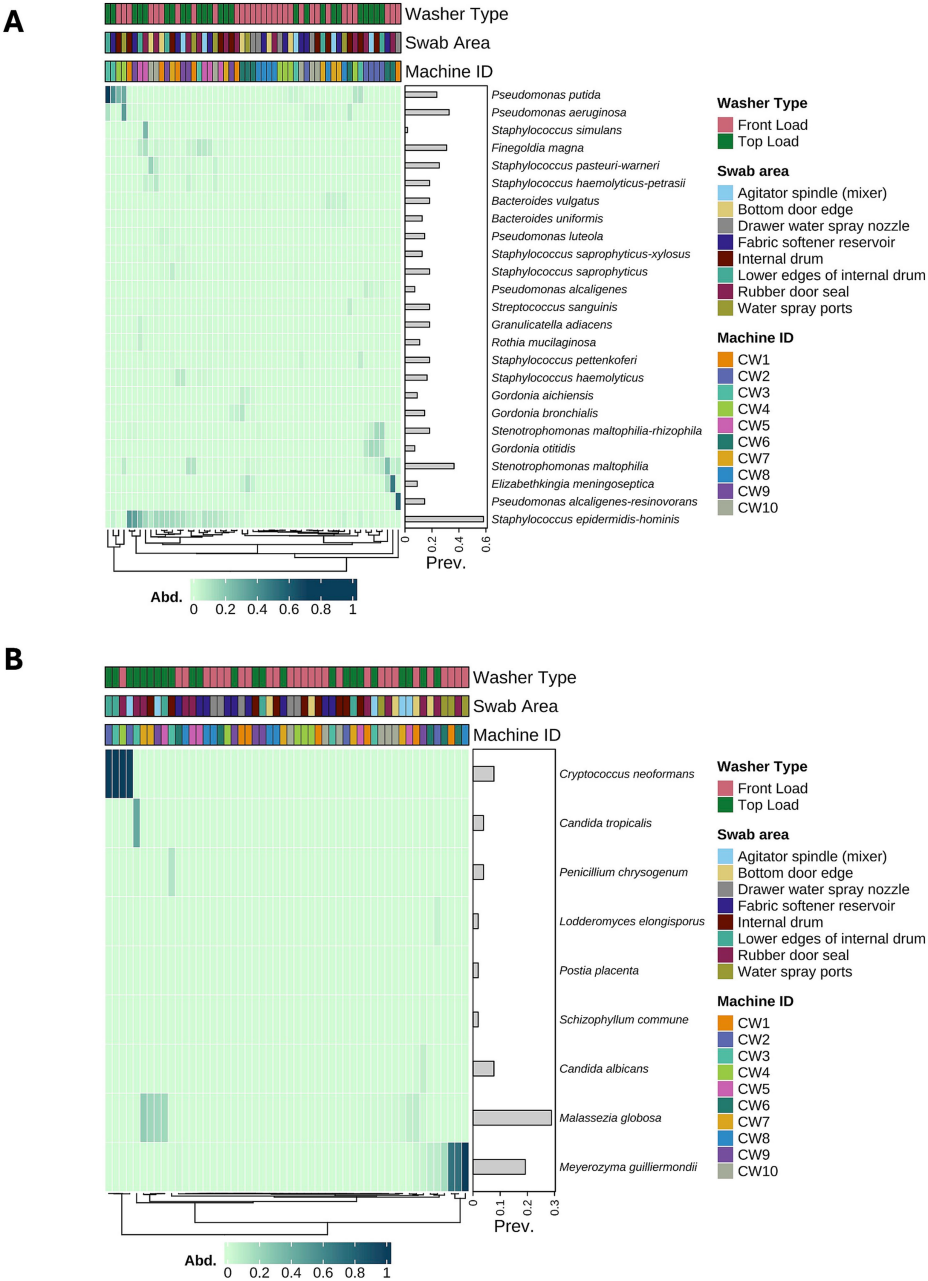
Potential pathogenic microorganisms in the surface swab samples. **(A)** Hierarchical clustering heatmap of the 25 most abundant potential pathogenic bacterial species in surface swabs, showing that opportunistic pathogens are present but differ in prevalence across machines and surfaces. **(B)** Hierarchical clustering heatmap of potential pathogenic fungal species illustrating the occurrence of known opportunistic fungi on machine surfaces.

To identify potential pathogenic microbial species in our dataset, we used the framework established in the Microbe Directory (Sierra et al., 2019), which includes a score predicting the potential likelihood of a microorganism being a pathogen. We refer to the bacterial and fungal taxa identified with pathogenicity scores as opportunistic pathogens, reflecting that most are not obligate disease-causing organisms but may cause infections under favorable conditions (e.g., in immunocompromised hosts or with high inoculum exposure). Upon closer examination of our results, we observed the presence of potentially pathogenic bacteria (score of 2) in some samples, including *Pseudomonas putida* and *Pseudomonas aeruginosa* (Figure 5A). In contrast, among the most prevalent taxa, but not necessarily highly abundant in all samples, we observed the presence of *Staphylococcus epidermidis*-*hominis*, an organism commonly present in the human skin microbiome. No pathogenic fungal species were detected. Only potential pathogens were present in the samples, including *Cryptococcus neoformans*, which had a high relative abundance in a subset of the samples (but with a low prevalence across the dataset), and *Meyerozyma guilliermondii*, another potential pathogenic fungal species, which was highly present in a subset of the samples (Figure 5B).

**Figure 6:**
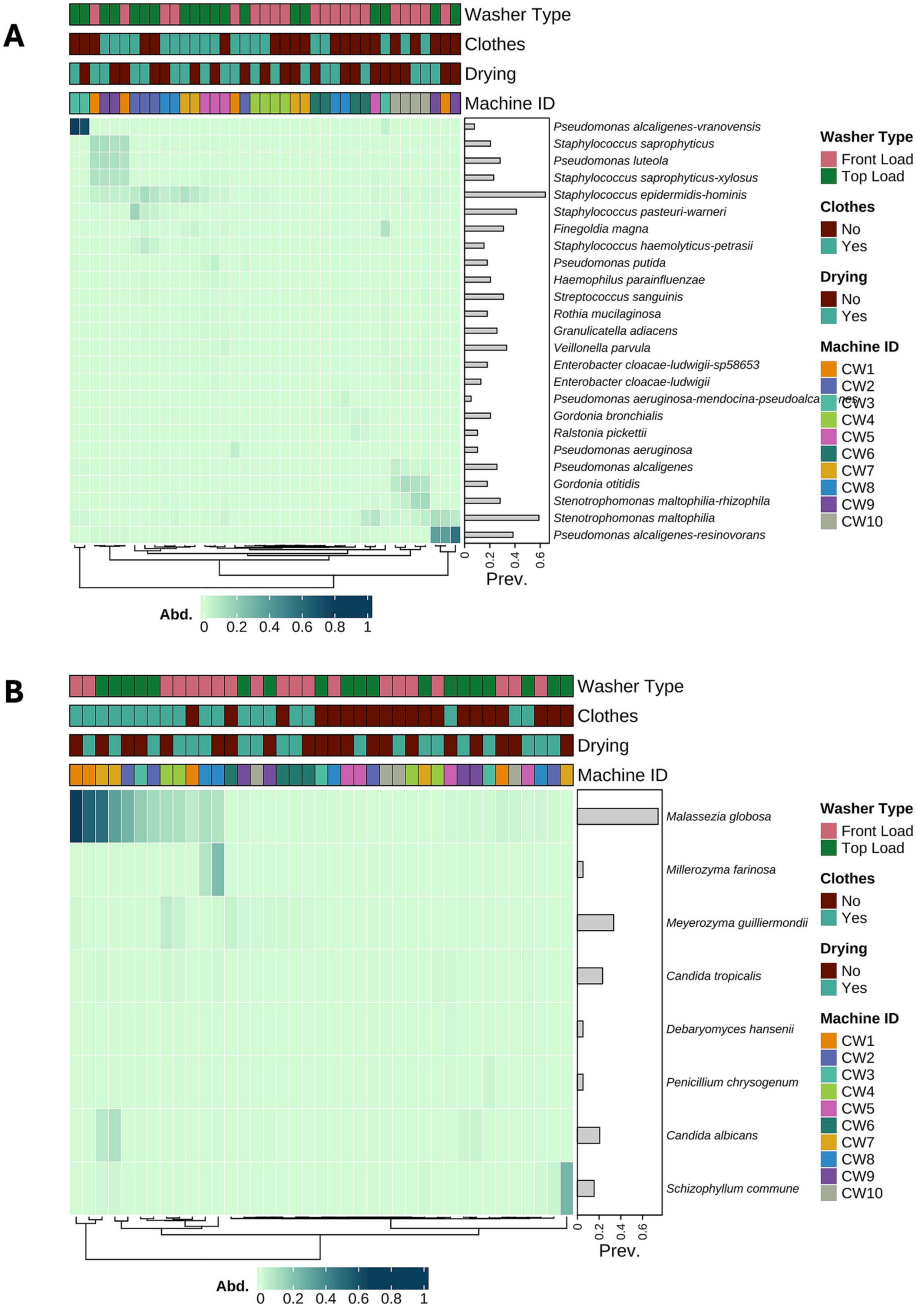
Potential pathogenic microorganisms in the washcloth samples. **(A)** Hierarchical clustering heatmap of the 25 most abundant potential pathogenic bacterial species in washcloths, demonstrating the transfer of opportunistic bacteria into fabrics. **(B)** Hierarchical clustering heatmap of potential pathogenic fungal species in washcloths, revealing the persistence of opportunistic fungi following washing cycles.

In the case of the washcloth samples, we observed a similar situation to that of the surface swabs. No pathogenic species (score of 3) were detected in any of the samples, and most of the bacterial and fungal species detected had the potential to be pathogens or were members of the human skin microbiome. For bacterial species, some samples had a high relative abundance of *P. alcaligenes*-*vranovensis* and *P. alcaligenes-resinovorans*, which had a prevalence of nearly 40% in all samples (Figure 6A). In contrast, among the most prevalent bacterial species in the washcloths, we detected *S. epidermidis-hominis* in most of the samples but with a low relative abundance across the dataset, and *Stenotrophomonas maltophilia,* a potentially pathogenic bacterium, which was present in nearly 60% of the samples. In the case of fungal taxa, no pathogenic taxa were detected in the washcloth samples, only potentially pathogenic taxa. *Malassezia globosa* was the most prevalent fungal group, present in nearly 60% of the samples, with a high relative abundance in a subset of the samples (Figure 6B).

## 4 Discussion

This study demonstrates that household washing machines contain diverse, user-specific microbial communities that persist across different machine designs and sampling sites. Front-load machines exhibited significantly higher microbial loads than top-load machines, likely due to design features that promote water retention and microbial growth. Although our sample size (n = 10; five front-load and five top-load machines) was modest, it was consistent with prior studies and provided a valuable foundation for advancing research in this area (Callewaert et al., 2015; Jacksch et al., 2021; Whitehead et al., 2022). These findings underscore the need for larger-scale investigations to validate and extend the patterns observed in this study, ultimately contributing to a more comprehensive understanding of the microbiomes of household items and their potential health implications.

This study has several limitations. Washing machines were selected through convenience sampling, which may introduce bias and limit the representativeness of the results. The small sample size (*n* = 10) restricts generalizability, despite its alignment with other similar studies. Household cleaning practices and products utilized were not fully controlled and could have influenced the microbial community structure, as could the quality of the input water used in the washing process. In addition, we did not collect information on household demographics, cleaning routines, or pet ownership, which could influence the microbial composition of machines. Although culture-based counts were performed for wet and dried washcloths, individual viable isolates were not identified, whereas DNA metabarcoding captured DNA from both viable and non-viable cells. Future studies should address these aspects by collecting information on household health conditions, incorporating water quality assessments, and providing detailed accounts of washing practices and host-related factors to build a more comprehensive understanding of the washing machine microbiome. In addition, the incorporation of culture-based identification or viability PCR methods would provide a clearer picture of microbial survival on different surfaces and with different cleaning routines.

### 4.1 Surface swabs

Our research revealed the pervasive presence of microbial communities, comprising bacteria and fungi, on various surfaces in washing machines, regardless of the area sampled or the machine design (front-load or top-load). The bacterial levels in swabs collected from all locations of the front-load washing machines were higher than those in the top-load machines, except for the drum wall. We theorized that these differences could be explained by the fact that front-load machines retain residual water in the drum, creating an environment that may promote or support microbial growth, in contrast to top-loading machines, which, in general, drain more completely.

Most bacterial genera identified in the samples had been previously identified in other similar studies, such as members of Proteobacteria, Actinobacteria, and Bacteroidetes (Nix et al., 2015; Jacksch et al., 2019). Among the most common bacterial taxa identified in the surface swabs were those belonging to the genus *Micrococcus*, which were found in a substantial proportion of the samples and were highly abundant in a subset of them (associated with two different users). This genus of microorganisms is commonly present in the skin microbiome, as well as in the environment. It has been detected in synthetic clothing fabrics, which are thought to play a role in odor development (Callewaert et al., 2014). This finding suggests that the individual habits of washing machine users may influence the microbial community composition. Furthermore, this study underscores the importance of adopting a more holistic approach that considers the habits and lifestyles of users, the type of clothing commonly used, and the machine itself.

Further examination at the genus level (Figures 1B,C) revealed that the similarities among the samples were primarily attributed to the machine owner rather than other variables. This finding was supported by the beta diversity analysis (Figure 4), which revealed a significant separation of the community based on the machine owner and, to some extent, the type of machine sampled, but no correlation with the area of the machine that was swabbed. This suggests that human activity is the primary driver of the types of microorganisms found in these appliances. Washing machine users have unique habits, such as dietary preferences, lifestyle choices, and the use of cosmetic products, which can influence their microbiome composition. This microbiome composition is reflected in the types of microorganisms present in the washing machines. This type of correlation has been previously observed in studies of the indoor environment, where the microbiome of each home was identifiable by its inhabitants (Lax et al., 2014).

Moreover, a recent survey of household washing machines found that the main contributors to microbial communities were the users and not the water source (Chen et al., 2024). The results of our study differ from those of previous reports, in which the bacterial community was associated with the swabbed area (Jacksch et al., 2019). These differences can be attributed to the high level of variability and dynamic nature of these communities. This highlights the variability in this type of study and the need for more standard analyses controlling additional variables, such as user habits, water analysis, products used, and the kind of machine, among other parameters.

In the case of fungal communities, we observed results similar to those of previous reports that examined fungal communities in washing machines (Zareshahrabadi et al., 2023). Cladosporium was one of the most prevalent fungal genera, present in all samples, and exhibited a high relative abundance in some swabbed areas. This fungal group can be found in indoor and outdoor molds, and some groups are also part of the human mycobiome (Seed, 2015). Similarly, other abundant groups are associated with the human mycobiome, such as *Saccharomyces* and *Malassezia species*. These observations, along with the differences from previous studies, highlight the need for more detailed observations that will allow for a more specific taxonomic and functional characterization of these communities in the future. This will enable differentiation between those originating from environmental sources (such as water or indoor air) and those originating from the user.

### 4.2 Washcloths

The findings from the washcloth samples highlight the influence of the user on the composition of the microbial communities. The most prevalent bacterial genera identified were *Acinetobacter*, *Micrococcus*, *Moraxella*, *Mycobacterium*, and *Pseudomonas*. Several of these taxa were also present in the surface swabs but are also commonly found on human skin. For instance, the detection of *Propionibacterium* in the samples is a clear example of human input, as it is one of the most common genera found in the human skin microbiome (Byrd et al., 2018). Similarly, the most prevalent fungal taxa were *Malassezia*, *Rhodotorula*, and *Cladosporium*. A combination of environmental and human inputs is likely, but a more in-depth analysis is necessary to determine this. Further studies should examine the skin microbiome of machine users to track taxa over time and space using microbial source tracking approaches (Knights et al., 2011; Shenhav et al., 2019).

The results of our study were unexpected in that machine drying had no significant impact on the viable bioburden of the laundered articles, nor did it result in any changes in the microbial community composition, as indicated by the beta diversity analysis. However, the presence of clothing had a notable impact on these communities. This finding has important implications for handling clothes before they are placed in a washing machine, as they can be a significant source of biological contaminants. Prior research has explored the role of clothing as a vector for the transmission and movement of microorganisms in hospitals. However, to our knowledge, the impact of clothes on the washing machine microbiome in conjunction with factors such as machine type and drying has not been studied.

### 4.3 Opportunistic microorganisms

We analyzed the pathogenic potential of microorganisms identified in our dataset, focusing on a subset of ASVs that could be classified at the species level, and leveraging existing databases to quantify the pathogenic potential of these species. To this end, we applied a scoring system adapted from the Microbe Directory (Sierra et al., 2019), which integrates available information on virulence traits and pathogenic associations. Although this approach provides a standardized framework grounded in a carefully curated dataset, it has not yet been extensively validated across diverse contexts. The Microbe Directory has also been applied in studies of cleanroom environments (Danko et al., 2021), gut and oral microbiomes (Podlesny et al., 2022; Valles-Colomer et al., 2023), and environmental datasets (Kolokotronis et al., 2025), further supporting its utility. Our study extends the use of this approach to the washing machine microbiome and environment, which have not been previously examined. Future studies comparing this scoring system with similar published data would be valuable for further assessing its reliability and refining its application in environmental microbiome studies. The results indicated that all species identified in the dataset had pathogenic scores that suggested they were potential pathogens but not high-risk microbes. The pathogenic potential of an organism is influenced by various factors, including the presence of virulence and antibiotic resistance genes, the immune state of the individual who becomes infected, and the inoculum size (Casadevall, 2017; Smith and Casadevall, 2022). Further research is needed to fully quantify the health risks associated with the microbial species present in the washing machine microbiome of healthy individuals. In this study, we did not collect information regarding the health conditions of the volunteers, which represents a limitation of the study and an aspect that should be included in future work. Nevertheless, owing to the limitations of the current study, we did not identify highly pathogenic microbial species that pose a risk to human health. It is essential to consider that some users in high-risk groups, such as immunocompromised individuals, may need to take additional precautions to prevent potential health issues resulting from exposure. Our analysis also revealed the presence of various microorganisms typically found on human skin, such as *Staphylococcus epidermis* and *Malassezia globosa*, as well as different species of *Pseudomonas* (*P. putida, P. aeruginosa, P. alcaligenes*), *Staphylococcus* (*S. pasteuri, S. haemolyticus, S. saprophyticus, S. pettenkoferi, S. epidermidis*), and *Gordonia* (*G. aichiensis, G. bronchialis, G. otitidis*), among other taxa. All of these species can be found in the human skin microbiome (Byrd et al., 2018) and have the potential to be pathogenic under specific conditions.

The washcloth dataset indicates that laundry practices can be a source of opportunistic pathogens. This finding highlights the importance of adhering to proper hygienic laundering practices, particularly in households with immunocompromised individuals or during outbreaks of communicable illnesses, to prevent the transmission of microorganisms between clothing and machines. Beyond household contexts, it is essential to note that public laundromats exhibit a higher microbial burden due to their shared use by multiple users and textiles, resulting in increased microbial turnover and potential exposure compared with home laundering (Callewaert et al., 2015; Whitehead et al., 2022). Although documented cases of transmission are lacking, these contextual differences suggest that the risks may vary between domestic and public laundering environments, highlighting the need for further comparative studies.

## 5 Conclusion

Washing machines have been a staple of modern life for more than 100 years. They are ubiquitous in millions of households worldwide (Pakula and Stamminger, 2010) and play an essential role in our laundry habits. Understanding the potential for microbial colonization and contamination of these artifacts is crucial for designing improved appliances, developing novel cleaning compounds, and designing improved cleaning strategies. This study revealed that household washing machines contain significant microbial loads and user-specific communities, with front-load machines showing a higher bioburden than top-load machines. The persistence of microorganisms after standard washing and drying cycles, combined with the detection of potential opportunistic pathogens, suggests that current household laundering practices may be insufficient for eliminating all microorganisms from textiles. These findings have important implications for vulnerable populations and support the implementation of supplemental sanitization strategies in household laundry.

Additionally, the study demonstrated that consumer washing machines are capable of holding a diverse community of microorganisms (often unique to the household), some of which may include opportunistic pathogens, with the capacity to produce biofilms (Gattlen et al., 2010). This suggests that current laundering processes and standards may need to be more effective in eliminating potential pathogens from laundered articles and the surfaces of washing machines themselves. This supports the use of supplemental strategies and/or products (such as laundry sanitizers) to enhance the efficiency of the process, particularly in households with immunocompromised individuals.

It is well established that bacterial malodors persist and are primarily a source of concern within the damp, soil-laden environment of household washing machines. Laundering practices that incorporate actions such as the use of high heat, bleach, and other laundry sanitizers and additives (as detergent alone is not sufficient in reducing the overall bioburden), as well as appropriate sanitizing/cleaning of the internal areas of the machine itself, can all potentially aid in reducing not only the presence of opportunistic pathogens but also the microbial populations contributing to machine and laundry malodors.

From a consumer perspective, our findings suggest that although most microorganisms detected in washing machines are opportunistic rather than obligate pathogens, they can persist through typical laundering processes and may be transferred between machine surfaces and laundry items. For healthy individuals, the risk of infection is likely low; however, for immunocompromised individuals or households with ongoing infectious illnesses, additional precautions should be taken (Bockmühl et al., 2019; Abney et al., 2021). Simple measures, such as regular cleaning of seals and detergent drawers, occasional high-temperature or maintenance cycles, proper drying of machine interiors, and the use of supplemental laundry sanitizers when needed, can help reduce microbial persistence (Osta-Ustarroz et al., 2024).

Although these recommendations primarily apply to household contexts, it is also important to distinguish between household and public laundromat environments. Household washing machines are primarily exposed to the microbiomes of a limited number of users, whereas public laundromats are shared by many individuals, leading to a higher turnover of textiles and potentially greater microbial exchanges. Although no clear cases of microbial spread from laundromats to users have been documented, previous studies suggest that the microbial burden in public machines may differ from that in household machines because of more intensive use and a wider range of inputs (Callewaert et al., 2015; Jacksch et al., 2019; Whitehead et al., 2022). Therefore, our results primarily reflect the risks associated with home laundering, and further studies are needed to directly compare domestic and public laundering environments.

Building on these distinctions, additional studies are required to (1) quantify the risk of infection resulting from laundry processes, explicitly addressing the transfer of opportunistic pathogens from clothing, washing machines, and other high-risk areas, providing a better understanding of the transmission routes between microbial sources; (2) determine the population levels of these pathogens necessary for infection; and (3) determine their survival rates after drying, particularly during periods of illness within the household.

## Data Availability

The data presented in the study are deposited in the NCBI repository, accession number PRJNA1337138.
