# Supplementary figures and images for: Microbial cross contamination in household laundering and microbial ecology of household washing machines
Source: Front Microbiol. 2025 Oct 17;16:1667606. doi: 10.3389/fmicb.2025.1667606 (PMC12575129; doi:10.3389/fmicb.2025.1667606)

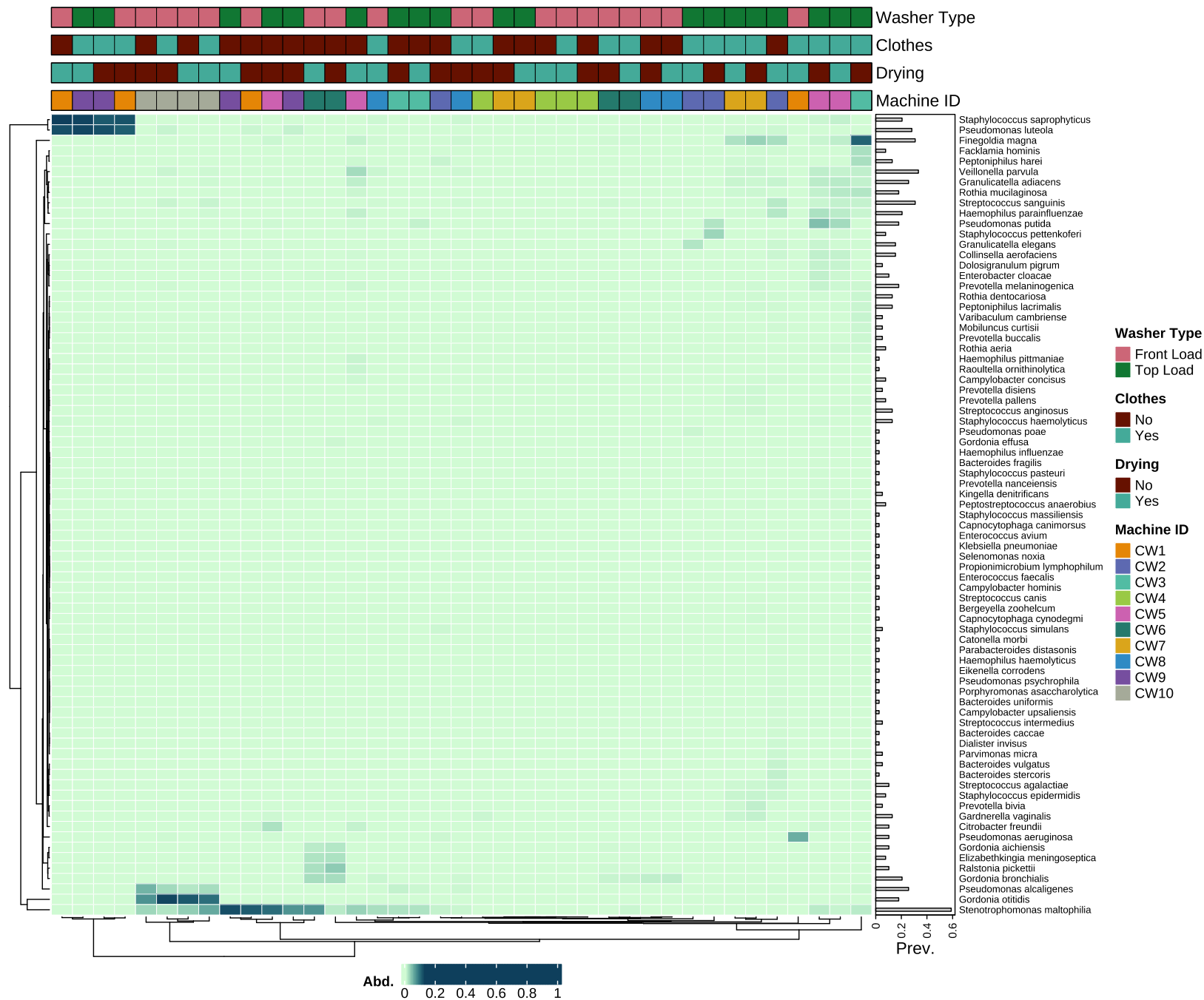

Supplement: Supplementary file 1 [file Supplementary_file_1.pdf]
